# Mixed-Matrix Membrane Fabrication for Water Treatment

**DOI:** 10.3390/membranes11080557

**Published:** 2021-07-23

**Authors:** Tawsif Siddique, Naba K. Dutta, Namita Roy Choudhury

**Affiliations:** Chemical and Environmental Engineering, School of Engineering, RMIT University, Melbourne, VIC 3000, Australia; s3642366@student.rmit.edu.au or

**Keywords:** membrane, mixed-matrix membranes, MMMs, fabrication, membrane fouling, nanomaterials, phase-inversion process, interfacial polymerization, electrospinning

## Abstract

In recent years, technology for the fabrication of mixed-matrix membranes has received significant research interest due to the widespread use of mixed-matrix membranes (MMMs) for various separation processes, as well as biomedical applications. MMMs possess a wide range of properties, including selectivity, good permeability of desired liquid or gas, antifouling behavior, and desired mechanical strength, which makes them preferable for research nowadays. However, these properties of MMMs are due to their tailored and designed structure, which is possible due to a fabrication process with controlled fabrication parameters and a choice of appropriate materials, such as a polymer matrix with dispersed nanoparticulates based on a typical application. Therefore, several conventional fabrication methods such as a phase-inversion process, interfacial polymerization, co-casting, coating, electrospinning, etc., have been implemented for MMM preparation, and there is a drive for continuous modification of advanced, easy, and economic MMM fabrication technology for industrial-, small-, and bulk-scale production. This review focuses on different MMM fabrication processes and the importance of various parameter controls and membrane efficiency, as well as tackling membrane fouling with the use of nanomaterials in MMMs. Finally, future challenges and outlooks are highlighted.

## 1. Introduction

Membranes can be described as films that act as selective barriers between two adjacent phases that allow the transportation of substances from one compartment to another [1]. Membranes play a vital role in separation technology, as well as in energy applications. Membranes are mostly polymer-based, which is adjusted by their synthesis process for the separation of specific substances, and results in efficient cost-effective separation technology with high performance. However, polymer-based membranes have some limitations due to their unavoidable built-in disadvantages, such as poor chemical and physical resilience.

Mixed-matrix membranes (MMMs) are an important class of organic–inorganic nanocomposite membranes with dispersed nanoparticles in polymeric films. Mixed-matrix membranes are based on either classical porous fillers such as zeolites, porous silica and carbon molecular sieves, or nonporous fillers such as graphene oxide, which has the ability to modify the free volume of a polymer by altering the molecular packing of the polymer chains in the membrane. The typical features of nanoparticles, such as stability, surface-area-to-volume ratio, surface charge, etc. [2], make them excellent candidates for inclusion in polymers for biomedical and environmental applications, including conventional water-treatment processes [3].

In the field of functional membranes, the use of a wide range of nanoparticles and the combination of them with other engineered novel materials gives great scope for engineering the shape and structure of the membranes with the desired performance. As a result, the use of mixed-matrix membranes (MMMs) is under development, in which nanoparticles are used as the filler materials in the polymeric matrix of MMMs [4] for applications such as water filtration, gas separation, fuel-cell application, and pervaporation [5,6,7]. MMMs have been developed substantially as per their applications, and new types of applications of MMMs also have been introduced in the past decade by incorporating inorganic nanomaterials such as metal oxides, including zinc oxide (ZnO) [8], titania (TiO_2)_ [9], iron oxides (Fe_2_O_3_, Fe_3_O_4_) [10], zeolite [5], silica [11], carbon nanotubes [4], graphene [12], graphene oxide (GO) [13], and metal–organic framework (MOF) [14] as fillers in the polymer matrix.

**Figure 1:**
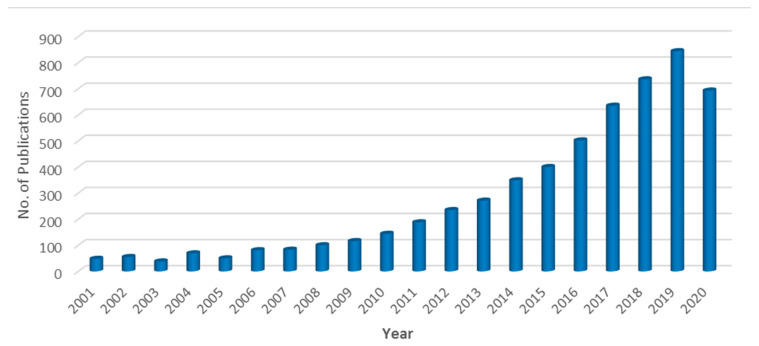
The number of publications each year since 2001 based on the keyword “Mixed matrix membrane” in the Web of Science database (data collected on 7 October 2020).

Currently, MMMs are fabricated using a wide range of fabrication processes based on the membrane materials and their applications. As the effective use of MMMs is increasing due to their various attractive properties, worldwide research on MMMs has experienced exponential growth, as indicated by the number of publications on MMMs in last 20 years (Figure 1).

Due to the significant roles of various fabrication processes on MMMs’ properties, the central focus of this review is the fabrication strategy of mixed-matrix membranes for water purification. We begin our discussion with the crucial issue of membrane fouling and ageing and the use of nanomaterials in membrane technology to address the issue, and then describe various fabrication strategies of MMMs, along with parameters that control membrane fabrication.

## 2. Membrane Fouling and Ageing: Major Challenges for Water-Separation Membranes

In general, membrane fouling occurs when undesirable particles, macromolecules, colloids, or salts are deposited on the surface of the membrane or inside the membrane’s pores. Membrane fouling can be subdivided into a few categories such as inorganic fouling, organic fouling, and colloidal/biocolloidal fouling, based on the membranes’ separation processes and the foulants’ chemical properties [15,16].

Inorganic fouling occurs due to the higher concentration of inorganic salts, such as sulfates, carbonates of sodium, calcium, etc., mainly when their presence in the solvents is beyond the solubility limits and results in precipitation on the membrane surface or into the pores of the membranes [17]. Organic fouling occurs when irreversible and strong foulants like humic substances, proteins, and polysaccharides are deposited on membrane surfaces [18]. In the case of surface-water, brackish-water, and seawater treatment, the main organic foulant is natural organic matter (NOM) [19]. When the membrane fouling occurs due to the deposition of colloids and the suspension of the nanoparticles or microparticles, it is known as colloidal fouling. There are three types of colloids [20,21]: organic colloids such as natural organic matter, proteins, etc.; inorganic colloids such as SiO_2_, iron oxides, and hydroxides of iron and heavy metals; and biocolloids such as viruses, bacteria, and other types of microorganisms. Biocolloid-induced membrane fouling is also called biofouling [22], and is caused by a range of bacteria like Aeromonas, Corynebacterium, Bacillus, Flavobacterium, Pseudomonas, and Arthrobacter, and also by fungi like Trichoderma, Penicillium, and other eukaryote microorganisms [23]. As a result, this foulant layer affects the permeate flux in two different ways [24,25]: first by creating an additional hydraulic resistance that results in low water flux and membrane permeability at a fixed applied pressure, which can be overcome by applying higher pressure; and also by the formation of a porous cake layer inside the unstirred cake layer, resulting in a higher concentration polarization, which leads to higher solute concentration on the membrane surface, as well as an increase in the osmotic pressure of the membrane surface and a decrease in the membrane flux.

Thus, it is widely recognized that the adherence of organic compounds and biocolloids to the surface of the membrane is the key parameter for the fouling of the membrane, and this adherence ability of the foulants is influenced by hydrogen bonding, London–van der Waals attractions, and hydrophobic and electrostatic interactions [18,26]. From the above discussion, it is evident that the inhibition or minimization of the fouling process might be possible by preventing the adhesion interactions between the membrane and the foulant. This could be possible through the development of MMMs with appropriate physiochemical properties, which could combine an efficient separation process with lower membrane fouling.

Additionally, various hydraulic cleaning procedures have been introduced for reversing or reducing membrane fouling [27]. Membrane backwashing with clean water is a common practice for foulant removal. After repeated filtration and backwash cycles, some materials are adsorbed on the membrane surface and need to be washed by a cleaning agent like hypochlorite for ultrafiltration membranes, as they cannot be removed otherwise [27]. Long-term exposure to foulants and cleaning agents has been reported to irreversibly change the performance and characteristics of membranes; these irreversible changes are defined as membrane ageing [28]. The characteristics of membranes are mainly chemical composition, pore size, etc.; and membrane performance factors are fouling rate, clean membrane resistance, etc. The main limitation is that complete full-scale ageing studies need many years of observation and cannot be controlled rigorously [29].

### 2.1. Effect of Membrane Surface Properties on Fouling and Ageing

The interactions between a membrane and foulants are determined by the membrane’s surface properties such as hydrophobicity or hydrophilicity, surface charge, and surface roughness [30,31].

#### 2.1.1. Hydrophilicity and Hydrophobicity of Membrane Surfaces

Usually, a membrane’s hydrophilicity or hydrophobicity is evaluated with a wettability study using contact-angle measurement [32]. The commercial membranes are mostly fabricated from hydrophobic polymers with high thermal, chemical, and mechanical stability, including polysulfone (PSF), polyethersulfone (PES), polyvinylidenefluoride (PVDF), polyacrylonitrile (PAN), polypropylene (PP), polyethylene (PE), and polyamide (PA) [1]. These polymers exhibit a high contact angle, which leads to the adsorption of different solutes from the feed. It is established that a higher mass per unit area of hydrophobic solute is adsorbed by membranes with high contact angles than that by the membranes with a lower contact angle [33]. On the other hand, hydrophilic membranes attract fewer charged inorganic particles, microorganisms, and organic substances, and result in less fouling [34,35].

#### 2.1.2. Surface Charge

**Figure 2:**
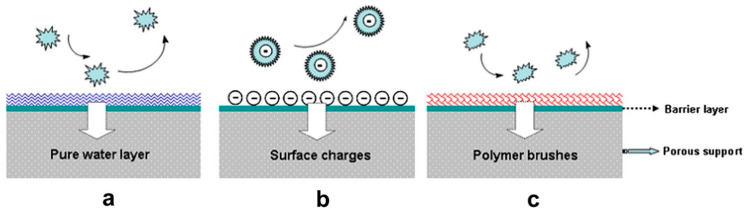
Schematic representation of various antifouling mechanisms with composite membranes: (**a**) thin layer of bounded water, (**b**) electrostatic repulsion, and (**c**) steric repulsion (adapted with permission from [39]).

In the case of charged foulants, membrane fouling can be controlled by the electrostatic charge of membranes. Membranes possessing the same charge as that of the foulants will reduce membrane fouling due to electrostatic repulsion occurring between the foulant and the membrane, which prevents foulant deposition on the membrane (Figure 2) [36,37]. Therefore, fouling can be reduced by incorporating ionizable functional groups on the surface of the membrane. For example, in protein filtration, when the protein is negatively charged at neutral pH, a negatively charged membrane surface could be a better choice [1]. Similarly, for organic compounds with a positive charge, the positively charged membrane surface is the solution for low membrane fouling [38]. So, low-fouling membranes could be fabricated and developed by considering the potential foulant’s charge on the membrane surface and inside the membrane pores from feed streams.

#### 2.1.3. Surface Roughness

Membrane fouling and surface roughness are strongly related to each other in nanofiltration (NF) and reverse osmosis (RO) membranes. Smooth and hydrophilic cellulose acetate (CA) RO membranes have less tendency toward colloidal fouling than hydrophobic and rough PA membranes [38]. Table 1 shows the relationship between surface roughness and relative fluxes for filtration of a sodium chloride solution containing silica particles with commercial NF (Osmonics HL, Dow-FilmTec NF-70) and RO (Trisep X-20, Hydranautics LFC-1) membranes. From the tabulated data of their flux and surface-roughness values, it is clearly visible that the flux decreased with the increase of surface roughness of the membrane during the filtration process. The increase in membrane surface roughness also led to an increase in the total surface area, resulting in more foulant attachment on the surface, and a ridge–valley structure also favoring the accumulation of foulants at the membrane surface. Using atomic force microscopy (AFM), Vrijenhoek et al. [40] showed that colloidal particles mostly accumulate in between the valleys of rough membrane surfaces, which results in valley clogging and causes lower flux and permeability than the membranes with smooth surfaces.

Considering all the above points, it is clear that the top membrane layer is the key area to control the fouling process, so the main goal could be the surface modification of the membrane to develop a low-fouling composite membrane by introducing polymer brushes and charged groups on the membrane’s surface, as well as hydrophilization and creating smooth surfaces, which would minimize the undesirable interactions between the foulants and the membrane surface for low or zero fouling of the membrane.

## 3. Mixed-Matrix Membrane Materials

### 3.1. Polymers

#### 3.1.1. Glassy and Rubbery Polymers

In water-treatment processes, various polymers have been used in MMMs; some polymers employed are rubbery (e.g., polyethylene oxide) [41], but most are glassy (e.g., aromatic polyamides, cellulose acetate, and polysulfone). Classifying membranes for water-treatment processes as rubbery or glassy can be complex, since they are operated under hydrated conditions and can absorb substantial amounts of water (i.e., ~10–50 vol% water) [42,43,44].

Recently, ion and water transport in glassy hydrated polymers has been reported, and has become a topic of interest in the membrane field [45,46,47,48,49]. Xie et al. measured water and salt transport in a disulfonated poly (arylene ether sulfone) copolymer (i.e., BPS-32) [45]. BPS-32 was synthesized in the potassium counter-ion form (K) and acidified to the acid form (H), either in solid state or in solution, and subjected to various ion-exchange steps and thermal treatments. Due to its relatively high T_g_ (278 °C), the membrane remained glassy upon hydration, and therefore its processing history had a profound impact on its water and salt transport properties.

More recently, Chang et al. prepared two chemically similar copolymers, rubbery 2-hydroxyethyl acrylate-co-ethyl acrylate (HEA-co-EA) and glassy 2-hydroxyethyl methacrylate-co-methyl methacrylate (HEMA-co-MMA), to probe the impact of polymer backbone dynamics on ion and water transport properties [48,49]. Both had similar and relatively low water contents (~8% by mass). However, the rubbery membrane had salt permeability coefficients roughly 2–3 times higher than those of the glassy membrane. In a later study, Chang et al. reported water dynamics and tortuosity in the same membranes over several length scales [49]. Using pulsed-field gradient nuclear magnetic resonance (PFG NMR), they measured water diffusivity as a function of diffusion encoding time. The longer the diffusion encoding time, the greater the length scale over which diffusion was measured. Water-diffusion coefficients decreased with increasing encoding time, plateauing at long times as water-molecule diffusion became increasingly hindered by the polymer segmental obstructions on longer length scales. The long-duration plateau value of water diffusivity was regarded as equivalent to the value observed in measurements of bulk-transport properties [49]. Salt solubility and diffusivity were measured via equilibrium and kinetic desorption techniques, respectively. Equilibrium water solubility was also measured. Using the solution-diffusion model, water and salt permeabilities were calculated from these data. Water and salt diffusivity and permeability were lower in the glassy polymer than in the rubbery polymer. However, water/salt selectivity was enhanced in the glassy membranes, corroborating the enhanced size sieving observed in their earlier study [48]. This result was mainly attributed to enhanced diffusivity selectivity in the glassy polymer, since salt solubility was similar in both polymers.

#### 3.1.2. Modification of Polymers

##### Chemical Cross-Linking

In many cases, membrane materials have reactive functional groups that can be linked through covalent bonds by applying a suitable cross-linker, which gives a remarkable scope of membrane fabrication using the chemical cross-linking process and for modifying polymers [50,51,52,53,54,55,56,57,58,59]. This chemical cross-linking method is used for a membrane’s mechanical strength enhancement or swelling reduction, as well as the increase of a specific solutes’ selectivity with better solvent permeability depending on the applications [60,61,62]. The cross-linking medium, the cross-linker’s concentration and molecular structure, and the reaction time/temperature mainly influence the cross-linking degree, as well as the charge density, which can be confirmed by Fourier transform infrared spectroscopy (FTIR) [50,58,63]. A polyvinyl chloride (PVC) membrane has been cross-linked with an activated-carbon loaded 4,4′-oxidianiline to prepare the MMM for separation technology [60].

##### Chemical Grafting

Chemical grafting on a membrane surface can be performed by growing or grafting another polymer onto the surface. The hydrophilicity, selectivity, and antifouling property improve due to the grafted polymer. There are a few approaches to produce the active sites that can prompt the commencement of the graft polymerization; for example, plasma, UV, and ion-beam irradiation [64,65,66].

**Figure 3:**
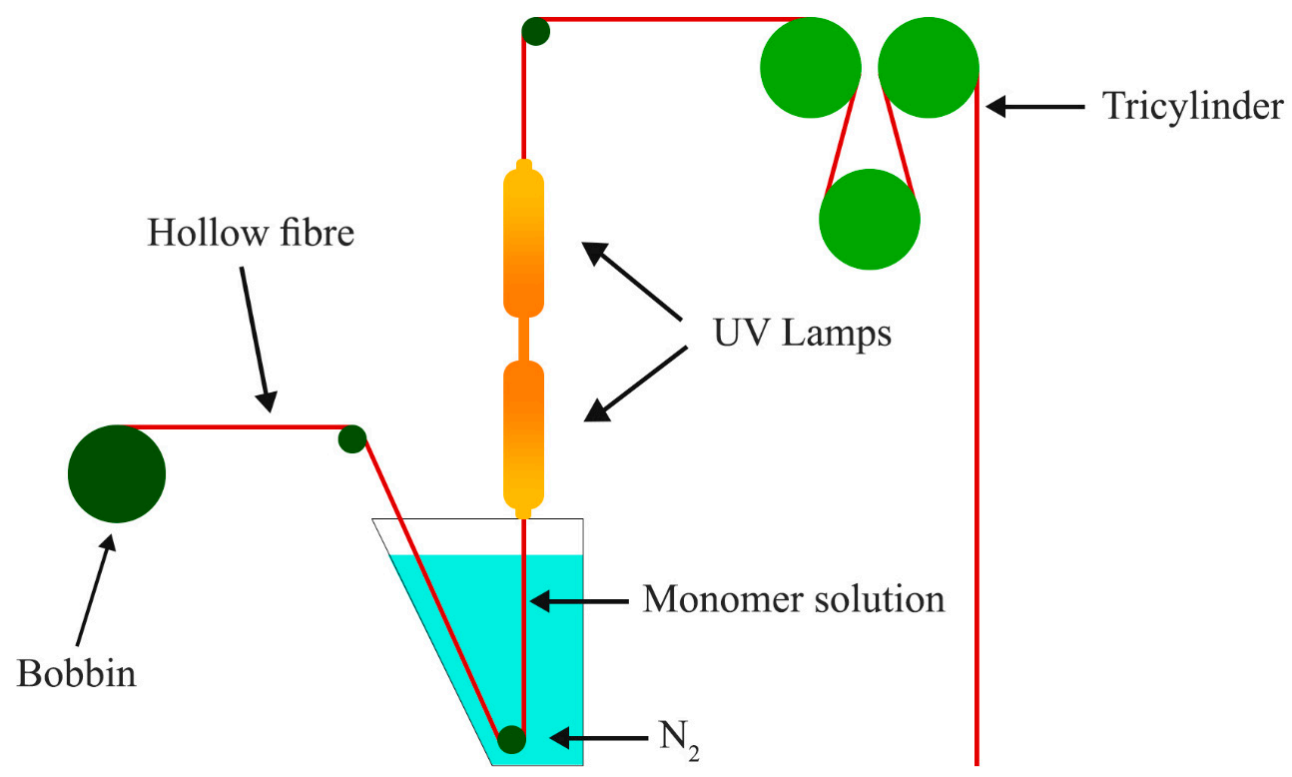
UV-photo-grafting setup for hollow-fiber membrane fabrication.

UV photo-grafting is performed on a polyimide membrane’s active surface to modify it so it is suitable for wastewater-treatment applications. The outer active surface of a polysulfone UF hollow-fiber membrane was reported to be achieved by UV grafting, in which sodium p-styrene sulfonate (monomer), N,N′-methylene bis acrylamide (cross-linker), and 4-hydroxybenzophenone (photo-initiator) were used. Figure 3 shows a UV-photo-grafting setup in which the support layers of hollow fibers are wetted by water and immersed in a monomer solution. At that point, the fibers pass through two UV polychromatic lamps [67].

Graft polymerization of a methacrylic acid monomer was reported to contribute to membrane hydrophilicity and negatively charge the membrane surface, as it could eliminate the disrupting endocrine chemicals and active pharmaceutical compounds [68]. Furthermore, the introduction of a redox reaction at the initial stage of surface grafting also offered hydrophilicity, and the redox reaction could be achieved in aqueous media at room temperature without any external activation [66]. Additionally, the concentration of the monomer needed to be higher due to the slow reaction kinetics of the redox initiation [69]. Commercial polysulfone (PSF) has been grafted by poly(polyethylene glycol) methyl ether methacrylate (PEG) side chains to improve the interfacial interaction with zeolitic imidazolate framework-8 (ZIF-8) nanoparticles to prepare the desired MMMs [70].

### 3.2. Nanoparticles (NPs)

Surface modifications of polymer membranes have led to various low-fouling membranes, and in some cases proved feasible for commercial purposes. However, the use of nanoparticles (NPs) in the membrane could be a better strategy for preparing low-fouling membranes in a simpler way with a long durability. The addition of a large variety of nanoparticles into the polymeric membrane has been extensively explored, leading to mitigation of membrane fouling with longer durability and high permeate flux [71,72]. The successful development of MMMs depends strongly on the polymer matrix selection, the inorganic filler, and the interfacial interaction between the two phases [73]. The selection of suitable types of inorganic filler and their surface modification dictates an MMM’s overall performance. Various surface-modification strategies have been used to maximize the interfacial interactions. The superior permeability and selectivity of inorganic membranes with the processability of polymeric membranes are combined in MMMs to achieve synergistic performance, in which the rigid, porous-type inorganic NPs provide desirable properties, and the polymeric phase enables the ideal membrane formation, hence solving the issue of brittleness inherently obtained in the inorganic membranes [74].

#### 3.2.1. Metal Oxides

Amongst various metal oxide nanoparticles, titanium dioxide (TiO_2_) is very attractive due to features like ease of preparation, stability, and commercial availability, and membrane fouling could be significantly reduced by introducing TiO_2_ into the polymer matrix of a membrane [9]. Additionally, the hydrophilicity and the free water fraction also increased with the deposition of TiO_2_ nanoparticles on the polymer membrane surface. Studies on the effect of various sizes of TiO_2_ nanoparticles in a hydrophobic polyvinylidenefluoride (PVDF) membrane revealed that the fouling activity of the PVDF membrane could be significantly improved using smaller nanoparticles [75,76], as this hydrophilic modification of PVDF membranes actually decreased the adsorption and deposition of hydrophobic organics on the membrane surface. For example, TiO_2_ in polyvinyl acetate not only decreased the membrane-fouling activity, but also improved the thermal stability, which was determined by the increase in the glass transition temperature [77].

Silica nanoparticles also showed the same trend in polyester urethane and polyether urethane-based membranes [11]. Silica nanoparticles have shown performance enhancement of polydimethylsiloxane (PDMS) membranes in pervaporation [78], resulting in improved selectivity of the membranes in pervaporation as the polymer chains became more rigid, and the polymer-free volume was also decreased.

Zinc oxide (ZnO) is used as the filler material in membranes for photo-degradation of organic pollutants and dyes in water and wastewater, and provides antibacterial properties [8]. It has also good electrochemical activity [79].

#### 3.2.2. Magnetic Nanoparticles

Nowadays, magnetic nanoparticles are considered as potential candidates for MMMs [80]. Iron-based magnetic nanoparticles have been studied for a vast number of environmental applications, as they also have the ability of bacterial inactivation [81,82]. Fe_3_O_4_ has been used as filler material in mixed-matrix membranes due to its attractive features for various applications such as oil–water separation [10], dye and magnetic-particle removal [83], etc.

#### 3.2.3. Carbon-Based Nanoparticles

Carbon-based nanomaterials are also considered as an efficient family of filler materials for MMMs due to their improved chemical and mechanical properties and cost-effectiveness. Among them, graphene oxide (GO) has been explored extensively as a filler material in the polymer matrix for the fabrication of polymeric nanocomposite membranes [84,85,86,87]. GO is a two-dimensional material with one-atom thickness, resulting in ultrafast water transport across the GO nanocomposite membrane as it forms interconnected nanochannels [88]. The functional groups such as hydroxyl (―OH), carboxyl (―COOH), epoxide, and C=C on the GO surface offer excellent hydrophilic, antifouling, and antibacterial properties [12,89,90,91].

#### 3.2.4. Zeolites

Mixed-matrix membranes with zeolite fillers have attracted attention due to their excellent advantages, such as high permeability and improved selectivity [92]. Zeolite–MMMs could be considered ideal for the purification industry, since they combine the properties of a polymeric matrix and zeolite inorganic fillers [93]. Nevertheless, only a few studies have been performed on zeolite–MMMs for water treatment; it was determined that the size of zeolite should be designed to match the expected polyimide active film thickness, thereby providing a preferential flow path through the nanochannels of zeolites [94,95]. Natural zeolite can readily form a suspension to coat the membrane as a support [96]. In another study by Damayanti and coworkers, zeolite-based membranes demonstrated excellent performance and high efficiency for removal of micro-pollutants for laundry-wastewater treatment [97]. Membrane performance was measured based on the flux and rejection values. They studied the superior ability of zeolite membrane to treat laundry wastewater as determined by turbidity measurements and phosphate removal as the two significant parameters. More importantly, another advantage of zeolite-based nanomembranes is that such membranes show an enhanced hydrophilicity when zeolites are used, since they are hydrophilic in nature, which in turn contributes to the enhanced removal of pollutants from wastewater. In addition, zeolite membranes showed improved separation performance and antifouling properties, and the structure and surface properties of the membrane’s thin-film layers were modified [98,99].

#### 3.2.5. Metal–Organic Frameworks (MOFs)

Metal–organic frameworks (MOFs) are a unique family of nanoparticles used with membranes for the enhancement of their separation performance, as well as in pervaporation to recover the bioalcohols [14]. MOFs decrease the ageing of the MMMs due to their good compatibility and interaction with the polymer matrix, which results in restrictions of chain mobility (one of the main causes of ageing) [100]. MOFs include ZIF-8 [101,102,103], HKUST-1 [103,104], and UiO-66 [100,105,106,107], mostly either as cast or modified [108]. MMMs with inorganic fillers or nanoparticles often have weak polymer−filler interfaces due to the lack of compatibility between the two components, which can create an adverse effect. MOFs containing organic functionality in their bridging ligands can potentially interact favorably with the organic functionality in polymers. However, the organic functionality does not completely eliminate this compatibility issue due to the rigid, crystalline nature of MOFs. Therefore, strategies to improve interfacial interactions, such as chemical and physical interactions, pre- and post-synthetic modifications to MOF ligands, chemically functionalizing the polymer, and employing cross-linking-type reactions to tether the MOF frameworks to the polymer, have been pursued [77,78,79,80,81,82,83,84,85].

Nanoparticles are also incorporated in membranes for pervaporation applications. As an example, for ethanol dehydration, phosphotungstic acid (H_3_PW_12_O_40_) nanoparticles were added in a sodium alginate/poly(vinyl pyrrolidone) polymer blend [109]. Silica nanoparticles have shown performance enhancement of polydimethylsiloxane (PDMS) membranes in pervaporation [78], resulting in improved selectivity of the membranes in pervaporation as the polymer chains become more rigid, and the polymer-free volume was also decreased.

There is a large body of work using nanoparticles and their surface modification, leading to better properties in many fields, as the general trend of using nanoparticles is to improve and maintain the permeability of liquids and gases and to enhance the desired separation of the membranes. However, the mechanism behind these results was not studied extensively. It is noteworthy that the nanoparticles in the matrix influence the morphology and free volume of the membranes. So, the nanoparticles are used in the membrane for their performance enhancement, and the fabrication of mixed-matrix membranes with nanoparticles will be discussed in the following sections.

#### 3.2.6. Loading or Addition of Nanoparticles in a Polymer Solution

MMMs are the combination of two phases: the polymer matrix and the filler material, such as NPs. Therefore, the mixing of NPs in the polymer matrix is an important part of MMM fabrication, as the homogeneous dispersion of NPs in polymer matrix needs to be ensured for good-quality membrane fabrication. To obtain this, preparation of a homogeneous solution of NPs and polymer is required, which can be done using one of the three established processes described below.

1. NPs are added to the solvent first and stirred for a predetermined time to prepare a well-dispersed solution, followed by the addition of a polymer in the dispersed solution [110,111,112,113,114,115,116,117,118,119,120,121,122,123,124,125,126,127].
2. The polymer is added to the solvent first and stirred for a specific time, and then the NPs are added to obtain the desired solution for MMM preparation [128,129,130,131,132,133,134,135,136,137].
3. The dispersed solution of NPs and the polymer solution are prepared separately in this process, and then the nanoparticle solution is added to the polymer solution [87,138,139,140,141].

Among these methods, the first and third methods are used for better distribution of inorganic particles because in a dilute suspension, the particles are prevented from agglomerating by a high shear rate during stirring, while the second method is commonly used for nanoparticle distribution in the polymer matrix [142].

## 4. Fabrication Processes of MMMs

**Figure 4:**
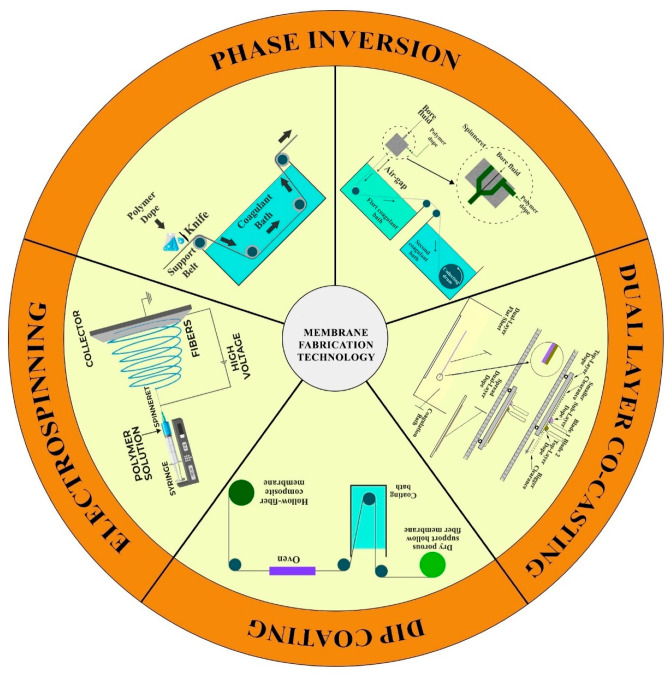
Various membrane-fabrication approaches.

Figure 4 shows the various membrane-fabrication processes that will be discussed in this review. The improvement in functional properties brought about by forming mixed-matrix membranes or nanocomposite membranes can be grouped in two categories: physical mixing and in situ synthesis [143]. The physical mixing method is very convenient to operate at a very low cost in large-scale production; as a consequence, it has been used extensively to fabricate nanocomposite MMMs. For any inorganic nanomaterials, the nanofillers and polymer dope typically are prepared independently and mixed using the solution, mechanical agitation, fusion, emulsion, etc. [144,145]. Inorganic particle deposition or direct coating onto the membrane surface could also be used to fabricate MMMs. Nonetheless, it is difficult to control the nanoparticles’ distribution on or in the polymer matrix during MMM fabrication through the direct mixing method of polymers and nanofillers. The interfacial adhesion of nanoparticles with the polymer can lead to larger aggregates during mixing, thus noticeably diminishing the advantages of the nano dimensions. In addition, polymer degradation upon melt compounding and phase separation of nanoparticles from the polymer phase is sometimes detrimental. The uniform dispersion of nanoparticles on or in the polymer matrix can be achieved by adjusting different processing parameters like shear force, time, and temperature, etc. [146], and the use of dispersing agents could be a promising way of obtaining a well-dispersed membrane [147].

**Figure 5:**
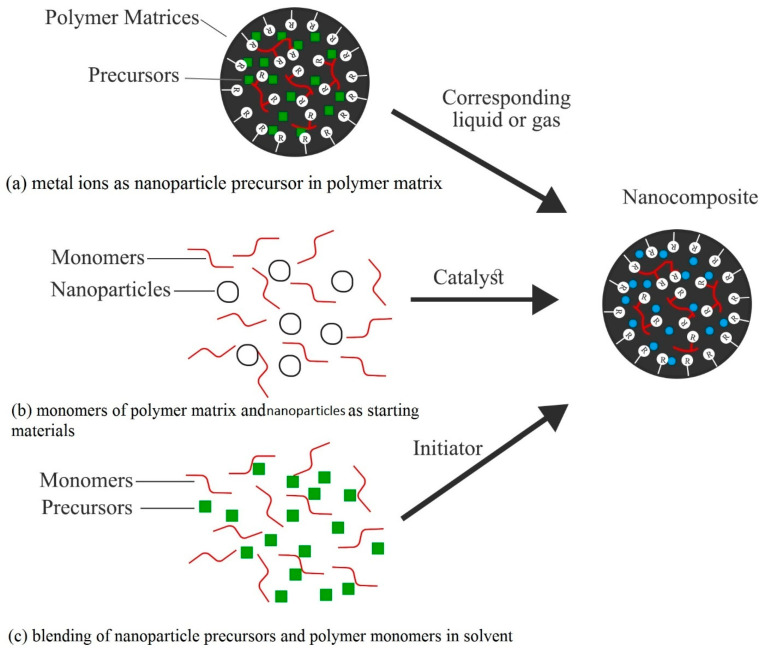
In situ synthesis process of mixed-matrix membranes.

The in situ synthesis process is also used for MMM fabrication, as some compounds like halides and sulfides can be easily and directly synthesized inside the polymer matrix. This in situ process has three categories, which are illustrated in Figure 5 in detail.

(a) A precursor solution of metal ions and polymer is exposed to the appropriate liquid or gas, which results in the in situ synthesis of nanoparticles in or on the polymer matrix with a uniform distribution [148,149,150]. A sol–gel method has been developed based on this for fabricating polyimide-based MMMs, in which titanium alkoxide solution was used as the precursor solution of TiO_2_ and modified by acetic acid [151].
(b) Another way is to start the synthesis with the solution of a monomer of the targeted polymer matrix and nanoparticles [152,153], in which polymerization takes place with the supplied desired catalyst at appropriate conditions just after the nanofillers dispersion into the monomer solution. This method allows the in situ nanocomposite synthesis of desired physical properties with a lower agglomeration tendency of the filler materials in the matrix.
(c) The other synthesis process is the combination of the above two, in which the precursor of desired nanoparticles and the monomers are dissolved in an appropriate solvent in the presence of an initiator for the in situ preparation of both the polymer and nanoparticles [154,155]. Based on this mechanism, a polyamide-based nanocomposite thin-film reverse-osmosis (TFN PA RO) membrane was synthesized from the dispersion of prepared zeolite in the trimesoyl chloride (TMC) solution [156].

Table 2 shows a list of membranes prepared according to various fabrication processes and their basic properties, and Table 3 compiles the merits and disadvantages of various fabrication processes. In the next section, we will discuss the main membrane-fabrication processes.

### 4.1. Phase Inversion Process

**Figure 6:**
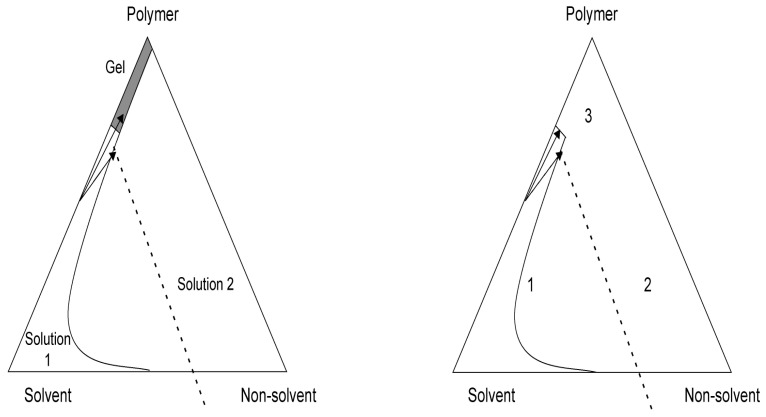
Phase diagram for the phase-inversion process.

Phase-inversion is the most popular method to form an asymmetric polymer membrane, and was first developed by Loeb Sourirajan in 1963 [175]. It offers several advantages over other membrane-fabrication methods such as material selection flexibility and the capability of making membranes with different pore sizes (between 1 and 10,000 nm) by varying the process parameters, solvent, and membrane material. The phase-inversion process is also called the phase-separation process, in which a homogeneous polymer solution is separated into two different phases, polymer-rich and polymer-poor, leading to two different layers of the porous structure. The mechanism of phase inversion primarily involves controlled transformation of a polymer solution to a solid state through liquid–liquid demixing, as shown in the ternary phase diagram of a polymer–solvent–nonsolvent system (Figure 6). Thermally induced phase separation (TIPS) and non-solvent-induced phase separation (NIPS) are the two approaches for the separation of a polymer solution. In TIPS, the polymer and solvent are mixed at a high temperature followed by cooling, which results in phase separation, whereas NIPS is a three-component process in which a non-solvent is used with the polymer and the solvent, and the main phase change occurs via the immersion of the polymer solution into the non-solvent [176]. During this immersion, the non-solvent is absorbed by the polymer solution and the volatile solvent is evaporated. An electrolyte membrane of PVDF and PAN polymers in which SiO_2_ was used as a nanofiller has been fabricated by phase inversion for lithium-ion batteries [159].

#### Membrane Fabrication through Immersion Precipitation

**Figure 7:**
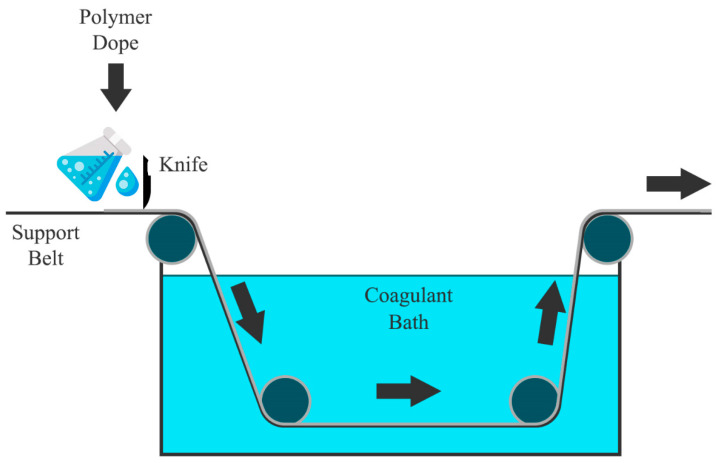
Illustration of membrane casting.

In the immersion precipitation method, a coagulation bath and a casting knife are used (Figure 7). The prepared homogeneous polymer solution is poured over a non-woven supporting mat, and then the dope is spread to a pre-defined thickness by using the casting knife. Afterward, the membrane is dipped into the bath. Before dipping in the bath, the casted dope is exposed to an ambient environment. The membrane property can be adjusted by controlling the temperature of the coagulant bath, as well as the exposure time in that bath, and the condition of the ambient environment. Although mostly water is used for the coagulant solvent, other non-solvents can also be used.

**Figure 8:**
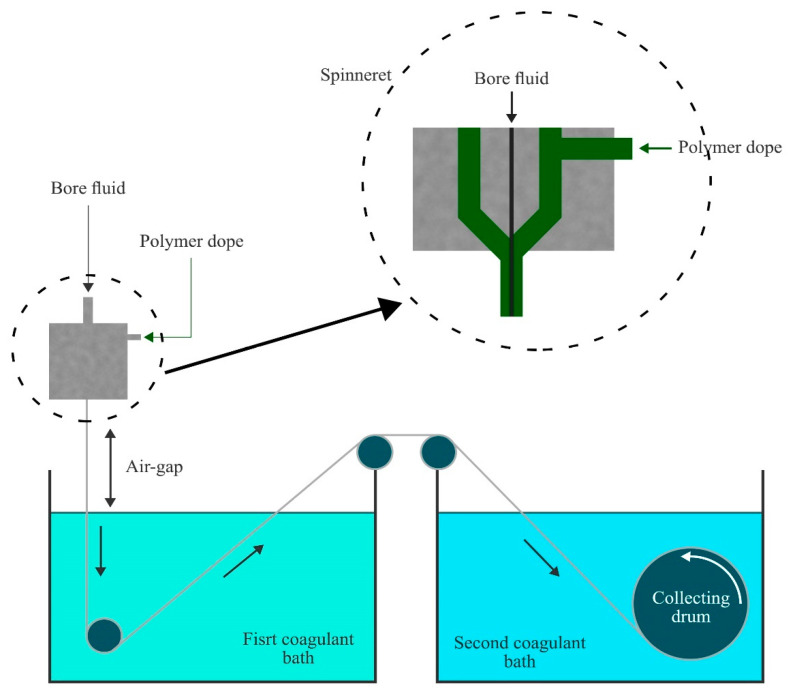
Spinning process of hollow-fiber membranes.

For hollow-fiber membranes [177,178], a bore fluid is required for hollow-fiber spinning as an internal coagulant. The process of hollow-membrane fabrication is complicated, as the phase separation occurs on both the inner and outer surfaces. A hollow-fiber fabrication process is illustrated in Figure 8. Extrusion of the bore fluid and the dope takes place simultaneously from the spinneret, and the pumps are used to control the flow rate. The developing fiber flows through an air gap and is finally immersed in the coagulant. A rotating drum is used to collect the final fiber at a constant speed, but it should be equal to or higher than the speed of free-falling fibers to avoid the coiling of the fiber. Finally, the solidified fiber is collected from the bath, followed by water soaking to remove the remaining solvent. Then the membrane is dried by freeze-drying or solvent exchange to avoid pore collapse during drying [177,178].

### 4.2. Interfacial Polymerization

Polyamide membrane development by interfacial polymerization has been recognized as the most regularly utilized method to form superior RO-like and NF-like active layers. Interfacial polymerization uses two exceptionally responsive monomers at the interface of two solvents that are immiscible with each other, one of which should be organic, and other of which should be inorganic/aqueous. There are two types of interfacial polymerization: (1) for drug delivery applications, micro/nanocapsules or micro/nanospheres are produced by dispersing one phase into another as tiny droplets using high-speed stirring [179]; and (2) the common process of introducing a continuous layer on a support, leading to a thin film [180,181].

A few types of monomers and prepolymers; for example, piperazine, N,N′-diaminopiperazine, and m-phenylenediamine for amine solution [182,183], and trimesoyl chloride, sebacoyl chloride, and iso-phthaloyl chloride for acyl halides solution [181] can be utilized for interfacial polymerization.

Mixed-matrix interfacial polymerization has been developed to insert nanoparticles throughout the polymer layer. The purpose is to improve the membrane’s performance. Super-hydrophilic zeolite nanoparticles are utilized to improve the water permeability with high rejection of salts [156]. Aquaporin-based biomimetic membranes have been fabricated with a similar process, resulting in high separation performances [184].

### 4.3. Multilayer Polyelectrolyte Deposition

**Figure 9:**
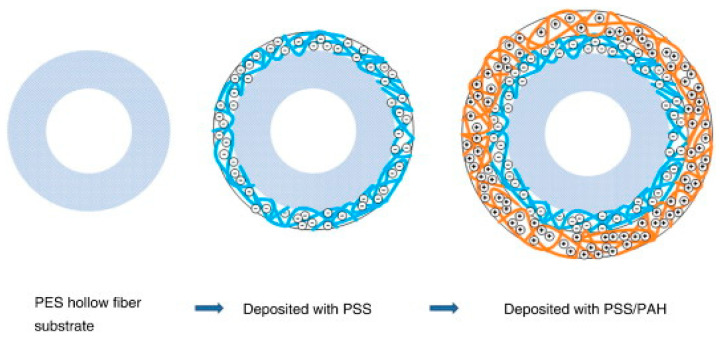
Schematic drawing of multi-layer polyelectrolyte deposition on the outer surface of a hollow-fiber membrane (adapted with permission from [185]).

**Scheme 1:**
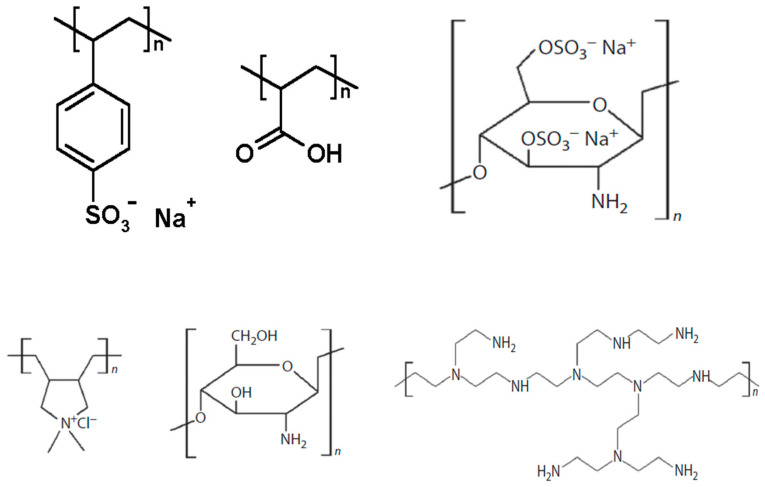
Commonly used polyanions and polycations for the development of active–selective layer: **Top:** polyanions, from left to right: poly(styrene sulfonate) (PSS) sodium salt, poly(acrylic acid) (PAA), sulfated chitosan (S-Ch); **Bottom:** polycations, from left to right: poly(diallyldimethyl ammonium chloride) (PDADMAC); chitosan(Ch); polyethylenimine (PEI).

Polyelectrolyte is a polymer containing electrolyte(s) groups in its repeating units. Polyelectrolyte shows charge properties when it dissociates in an aqueous solution or water. The driving force of multi-layer polyelectrolyte deposition on the membrane surface is the electrostatic interaction between the oppositely charged molecules. Scheme 1 shows various polyanions and polycations used for layer-by-layer formation of a polyelectrolyte complex multilayer (PEM). Figure 9 shows such a process, in which it is clear that the deposition of an aqueous polyelectrolyte solution on a porous substrate in the desired sequence could be a facile method of membrane preparation [185].

Multi-layer polyelectrolyte deposition is easy and adaptable for membrane preparation with thinner thickness and containing specific desired layers for high selectivity of the desired content. The function and structure of the layers can be different for specific applications based on the charge density of the polyelectrolytes and their molecular structures. Polyelectrolyte membranes are used in different applications such as forward osmosis [185,186,187], NF [188], ion exchange [189], pervaporation [190,191], and gas separation [192,193].

#### Factors Affecting Multi-Layer Polyelectrolyte Deposition

**Figure 10:**
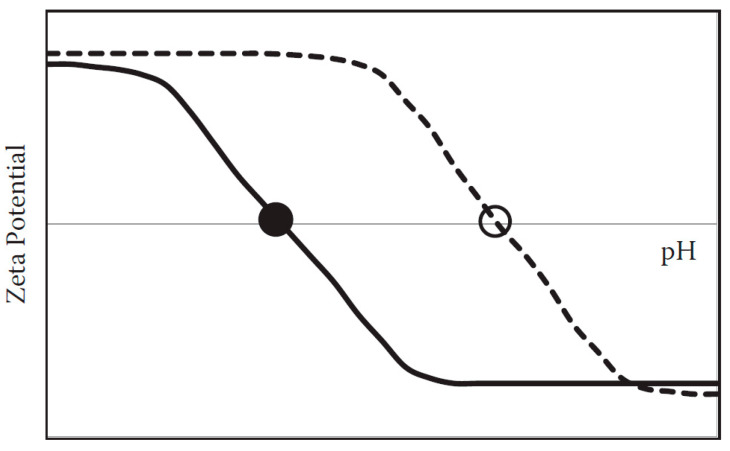
Plot of pH and zeta potential of polyelectrolyte (—) and substrate (---); ⚫, iso-electric point of polyelectrolyte; and ○, iso-electric point of substrate (adapted with permission from [196]).

The pH and ionic type of the polyelectrolyte multi-layer play a significant role in the development of a unique film of multi-layer polyelectrolytes. In the event that both permeable substrate and the polyelectrolyte are contrarily charged at a high pH and vice versa, the ideal pH utilized ought to be in the middle of the iso-electric point of the substrate and the polymer, as shown in Figure 10. Accordingly, inverse charges are conveyed by the substrate and the electrolyte [194]. An ultraviolet/ozone (UV/O_3_)-cleaned permeable alumina membrane with surface pore measurement of 0.02 µm is becoming attractive as a substrate for polyelectrolyte layer deposition because of its positive charges [195]. Plasma-treated/hydrolyzed polyacrylonitrile and cellulose acetic acid derivatization are negatively charged [186,187]. Furthermore, PES is also appealing as a supporting material in spite of the fact that it is neutral. Hence, the connection of polyelectrolyte layer depends on hydrophobic cooperation [185].

Ionic strength of the polyelectrolyte solution can be expanded by including salts. At high ionic strength, the electrostatic repulsion of the polymer chain diminishes with the polymer coils becoming denser, with the deposited layer in collapsed form instead of a flat conformation. Subsequently, it builds the thickness of the individual layer [197]. In the climate of incredibly high salt concentration, just a limited quantity of polyelectrolyte can be absorbed by the substrate because of the opposition to the more modest charged particles from the salts [194]. To improve the density of the polyelectrolyte layers, cross-linking could be utilized to enhance the layers’ stability. A cross-linking agent such as glutaraldehyde could be utilized in those cases [186,187]. Another parameter, the charge density of the polyelectrolytes, depends on the molecular structure and the degree of ionization of the polar groups. The charge density of the resultant multilayers, defined as the number of ionic groups per number of carbon atoms in the repeat unit of the polyelectrolyte complex/multi-layer [197,198] often guides the thickness. By adsorbing polyions from salt solutions of varying electrolyte concentrations, the layer thickness can be controlled over a wide range. In addition, consolidation of nanoparticles, such as silver on the active layer of a membrane, can also enhance the antifouling or antibacterial properties of the membrane. The layered structure of a multi-layer polyelectrolyte could improve the stability of the nanoparticles on the membrane surface [188].

### 4.4. Dual-Layer Co-Extrusion/Co-Casting

Improvement of composite dual-layer membranes is appealing, as beneficial properties of at least two polymeric materials can be consolidated for different applications. The material expense of the superior polymer can be decreased, and the polymer with extraordinary selectivity but poor mechanical strength can be reinforced, by consolidating them with an economical and strong polymer support layer [199,200].

Increasing uses of double-layer membranes include forward osmosis, gas separation, and NF membranes, which are made out of a thick selective layer supported by a porous polymer matrix [51,180,201,202,203,204]; and direct-contact membrane distillation, which requires an additional thin hydrophobic layer for wetting prevention and another hydrophilic layer for better water permeability [205].

**Figure 11:**
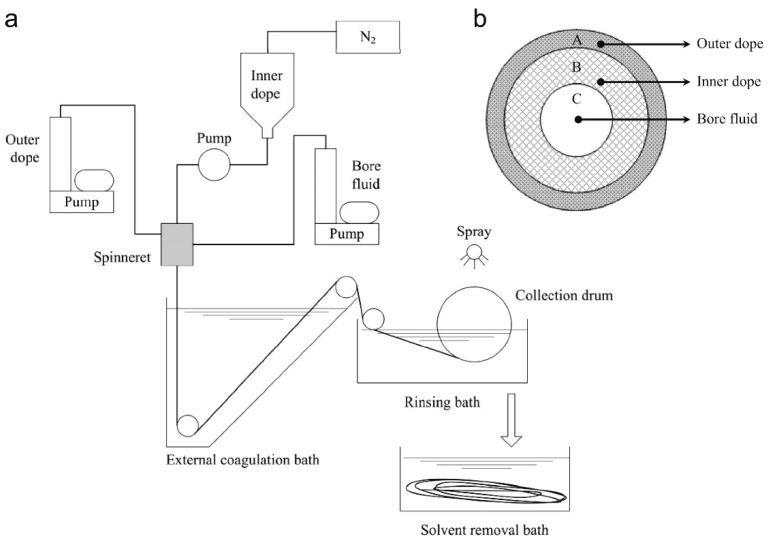

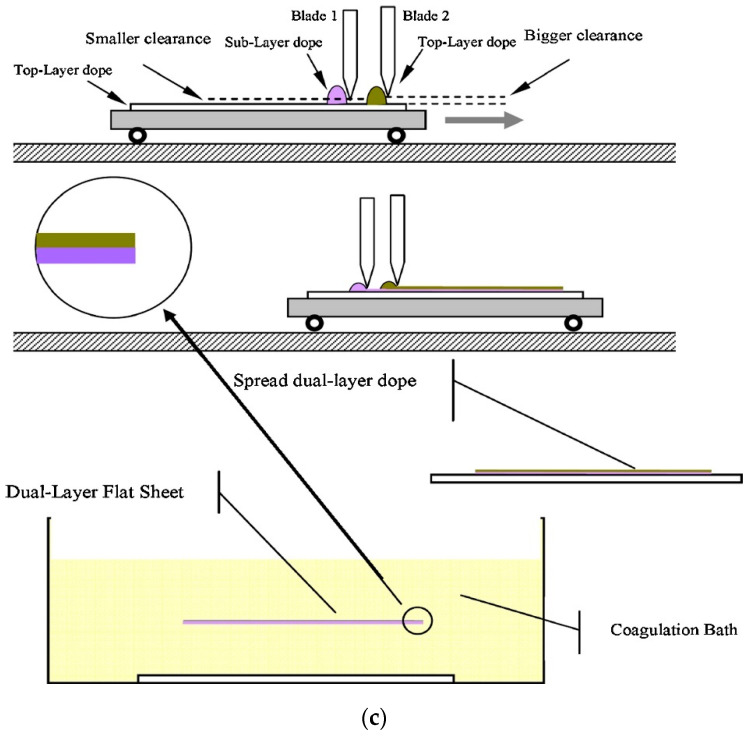
(**a**) Schematic diagram of a dual-layer hollow-fiber spinning process; (**b**) cross section of a triple-orifice spinneret (adapted with permission from [201]); and (**c**) fabrication process of a dual-layer flat-sheet membrane using a double-blade casting machine (adapted with permission from [202]).

Hollow-fiber [201,204] and flat-sheet [199,202] membranes are prepared by the dual-layer co-casting method on the basis of same principles, which are the casting of two different polymer solutions or a single-step co-extrusion. Synchronous development of the double-layer structure should be possible by utilizing a triple-orifice spinneret for hollow-fiber membranes, and a double-blade casting machine could be used to prepare the flat-sheet membrane by a co-casting process (Figure 11) [201,202].

Complexity arises while fabricating dual-layer membranes in either a hollow-fiber or flat-sheet configuration because of the involvement of many parameters controlling the thermodynamic properties and the energy of the phase change. This parameter control results in uniform cross-sectional morphology, as well as better lamination between the two layers of the synthesized membrane. The fabrication parameters can be divided into two categories: the chemistry of the polymer solution and the operating conditions. The chemistry of the polymer solution relies upon the polymer concentration and type, the solvent’s affinity to the polymer or coagulant, and the concentration and variety of non-solvent additives (or pore formers) [201,202]. Working conditions incorporate an air gap for hollow-fiber spinning, the evaporation time for flat-sheet casting, the composition and temperature of the coagulant, the temperatures of the polymer solution, and the operating temperature [177,199,201,206,207].

### 4.5. Dip-Coating

**Figure 12:**
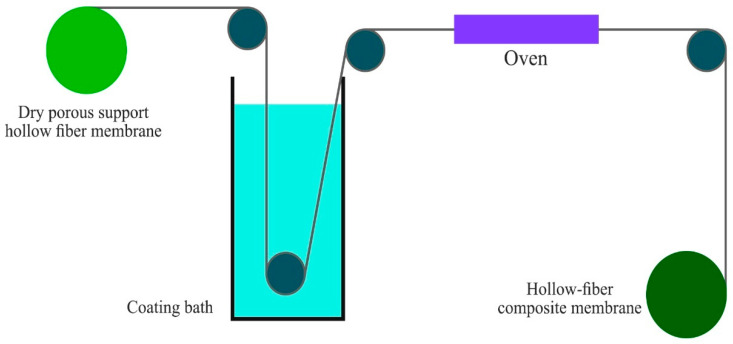
Hollow-fiber composite membrane fabrication by the dip-coating process.

In a dip-coating method, the membrane surface is coated by applying a polymer or organic materials. The polymer usually utilized as coating material should have some extraordinary properties; for example, it could be hydrophilic and negatively charged, and attach to the support layer easily. This group of polymers can be prepared by sulfonation; for example, sulfonated PES (SPES) and sulfonated poly(ether ether ketone) (SPEEK). The coating layer may upgrade the performance of the support layer; for example, by giving it a higher strength and better separation properties. Some basic properties should be taken into consideration while choosing the coating polymer; for example, the strength and stability of the polymer, layer-forming capabilities, easy solubility in solvents, cost, and cross-linking capability [208]. Three basic steps in the dip-coating process (Figure 12) are: (1) immersing a dry membrane in a coating solution, (2) permitting the coating material to interact with the substrate, and (3) drying the prepared membrane (Figure 12).

SPES has been used as the selective layer of NF hollow-fiber membranes by dip coating due to its capacity of ion exchange (limit of 0.8 meq/g) and antifouling activities. SPES conveys negative charges due to the presence of a sulfonic acid group in the main chain. The significant disadvantage of this polymer is that it can swell in water easily. When the polymer is dried, the structure of the layer becomes brittle [199]. In addition, NF hollow-fiber membranes have been prepared using PES as the substrate, followed by the dip-coating of SPEEK as the selective layer. The thickness of the coating layer generally relies upon the viscosity of the coating solution, which is impacted by temperature, grouping of the solution, and added substances. At a lower concentration, the viscosity of the solution is low, and as a result, the coating solution will infiltrate to the substrate pores [209].

### 4.6. Electrospinning

**Figure 13:**
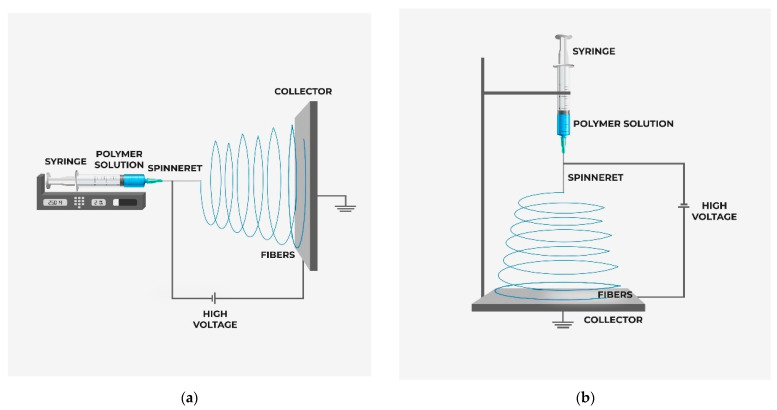
Schematic diagram of electrospinning setup: (**a**) horizontal; (**b**) vertical (adapted with permission from [212]).

Nanofibrous membranes are in high demand nowadays because of their scaffold structure, larger surface area, and interconnected porosity. Among different fabrication methods, electrospinning is attractive in developing nanofibrous membranes because of its scalability, simple design, and low cost [210,211]. Figure 13 shows a typical electrospinning setup.

**Figure 14:**
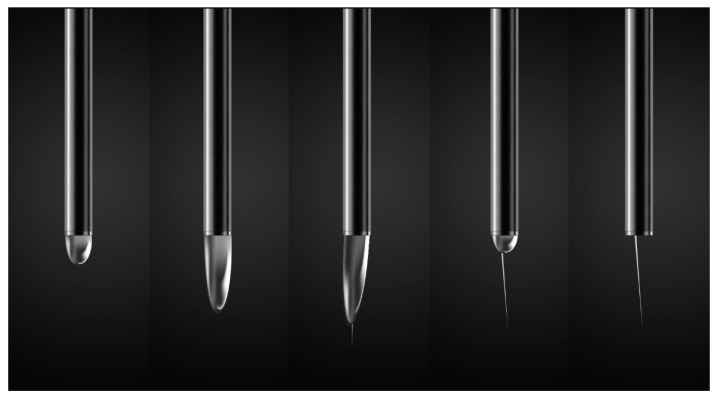
Formation of a Taylor cone with the increase of applied voltage.

Typically, the electrospinning system consists of a high-voltage power supply, syringe pump, syringe, needle, and a conductive collector where the fiber is gathered to make the membrane. Figure 13 represents a basic electrospinning system [212]. It can be classified as vertical and horizontal system based on the ordering of the spinneret. During the electrospinning process, the polymer solution is pumped at a suitable rate from the syringe to make small droplets at the tip of the spinneret. The voltage is supplied in the range of 1–50 kV from the high-voltage power supply, which results in charging of the droplet by the applied electric field, and eventually a solution jet is formed. The droplet is turned into a cone-shaped structure (Figure 14) to aim the solution jet toward the conductive collector. The threshold value of voltage causes the electrostatic force to overcome the surface tension of the droplets, which leads to the formation of jet from the cone’s tip. However, an appropriate viscosity is required for a continuous jet of solution by avoiding the Rayleigh instability, which causes breakup into droplets [213]. This jet becomes thinner and dries before being deposited on the collector in fiber form [214].

**Figure 15:**
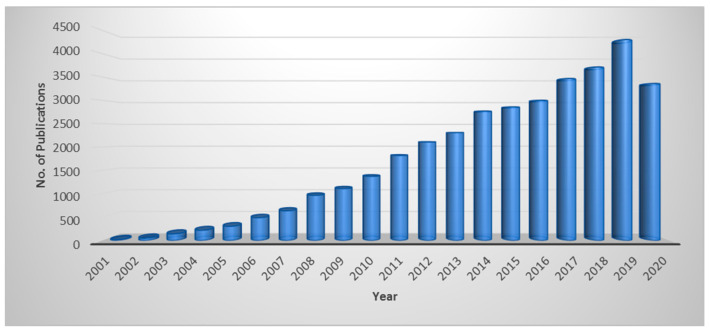
The number of publications each year since 2001 based on the keyword “Electrospinning” in the Web of Science database (data collected on 20 October 2020).

In 1930, Formhals illustrated the principle of electrospinning first, though the first patent was obtained in United States earlier (1902) [215,216]. Nevertheless, the electrospinning process received attention after 1990, but it was recognized globally within a short time to prepare the polymer-based nanofibers of different diameters down to a few nanometers. In the last decade, the number of publications on electrospinning is notable (Figure 15).

At present, the electrospinning process is more advanced than before, which allows a more controlled property by adjusting the process parameters. Eventually, the electrospinning method will become preferable in different fields of study, such as energy storage, separation and membrane technology, drug delivery, tissue engineering, and so on [217,218,219,220]. There is a drive to apply the electrospinning method in large-scale applications. Fortunately, Donaldson and Freudenberg [220] have successfully implemented electrospinning technology in making a filtration membrane.

**Figure 16:**
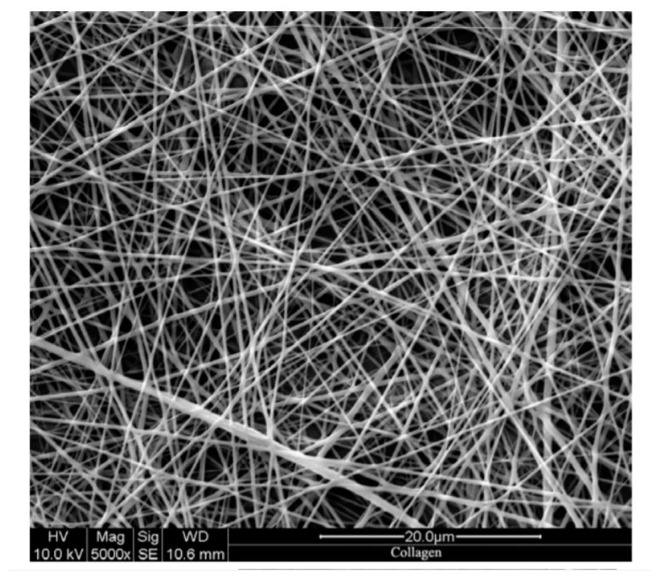
Scanning electron microscopy image of an electrospun polymer: a poly(acrylonitrile) non-woven nanofiber mat produced by electrospinning (adapted with permission from [221]).

The most interesting property of the electrospinning technique is the controllability of the fiber diameter by monitoring the variables such as solution concentration, loading of filler material, voltage, flow rate, temperature, and humidity [214]. A wide range of fiber diameters, from micron-sized to a few nanometers, can be achieved. Figure 16 shows a non-woven nanofibrous membrane of polyacrylonitrile [221].

The electrospinning method can be applied not only to polymers, but also to metals [222] and ceramics [223] for formation of micro- and nanofibers. However, polymers are mostly being studied, including mixed-matrix polymers containing polymer blends [224], drugs [225], and nanoparticles [226]. Although many polymers are being successfully electrospun into fiber, several polymer/solvent systems are very popular because of their suitable molecular weight, volatility, and conductivity of the solvent. This list includes polyamides [227], polyurethanes [228], polyester [229], poly(ethylene oxide) [230], polystyrene [231], poly(vinyl pyrrolidone) [232], poly(methylmethacrylate) [233], poly(vinyl alcohol) (PVA) [234], poly(lactic-co-glycolic acid) [235], polyacrylonitrile [236], and poly(caprolactone) [237], as well as bio-polymers such as chitosan [238], collagen [239], and gelatin [240].

In the next sections, the effect of properties of polymer solution and process parameter on the properties of electrospun membrane will be discussed. In addition, the roles of temperature and humidity are also mentioned.

#### 4.6.1. Effect of Intrinsic Properties of Polymer Solutions

The properties of a polymer solution largely control the structure of the nanofiber. Currently, a large number of studies have reported the role of solution viscosity, surface tension, concentration, and conductivity on nanofiber fabrication [241,242,243,244,245]. In the next section, the effect of these parameters will be described.

##### Polymer Concentration and Solution Viscosity

Several research reports showed that the structure and morphology of the electrospun membrane largely depend on the solution viscosity and concentration [214,246,247,248]. Polymer concentration profoundly influences the surface tension and viscosity of a solution, which eventually controls the development of nanofibers. The low-viscosity solution results in bead-on-string fibers. On the other hand, with increasing viscosity, the shape of beads is changed from globular to a spindle-like structure, which leads to the formation of a uniform fiber [229,249]. However, high viscosity also increases the diameter of the nanofiber. Therefore, it is required to optimize the threshold value to obtain a preferable fiber structure.

##### Electrical Conductivity

The spinnability of a polymer largely depends on the electrical conductivity of the dope solution, as the rheological behavior largely depends on it [250,251]. The category of solvent and polymer and the concentration of ionizable salts determine the electrical conductivity of the polymer solution [249]. Usually, a highly conductive solution forms a finer fiber and a wide range of fiber-diameter distribution [217,218]. In addition, increased electrical conductivity can help to form a stable Taylor cone that leads to producing a dense scaffold structure [252]. The conductivity can be enhanced by adding ions in the dope solution. Moreover, due to a higher charge density, the smaller ion can create a stronger elongation force on the jet [217,253,254,255]. Electrical conductivity can also be enhanced by adding a suitable acid with a higher dielectric constant, such as formic acid [256,257].

##### Surface Tension

Surface tension of the dope solution is an important parameter in tailoring the nanofiber structure. It can be adjusted by adding surfactants [254,258,259,260]. Lower surface tension forms a stable jet, and consequently, a uniform woven structure is formed. However, a higher amount of surfactant can cause other defects, such as a clustered structure.

##### Solvent

Solvent plays an important role in determining the morphologies of a nanofibrous membrane. During fabrication of a nanofiber, the solvent is continuously evaporated. Therefore, solvents with different evaporation and solubility rates can change the final structure of the nanofibrous membrane [254,257]. It has been reported that a solvent with low solubility is suitable for electrospinning. Spinnability–solubility maps were used to select a suitable solvent for the polymer [261].

#### 4.6.2. Effect of Electrospinning Process Parameters

The process parameters of the electrospinning technique such as flow rate, applied voltage and collector-to-spinneret distance play important roles in determining the quality of the electrospun membrane. In the following section, the effect of electrospinning parameters on the final product will be discussed.

##### Applied Voltage

The applied voltage determines the electrostatic force between the spinneret and the collector, and the charge density in the droplets [258]. The fiber diameter decreases with increasing voltage [252]. However, it may cause increased bead structure on the polymer net [262].

##### Electrode Distance

The distance between the spinneret and the collector defines the intensity of the electric field and the duration of the jet touching the collector. The distance should be enough to allow sufficient time for fiber elongation [263].The fiber elongation and solvent evaporation can be decreased by decreasing the distance, which leads to formation of a thicker fiber [264]. However, reduced distance also helps to stabilize the solution jet [265], while an inappropriate distance causes formation of beads [263].

##### Solution Mass Flow Rate

The study of the impact of flow rate on the quality of nanofibers has not been studied extensively. However, Megelski et al. [231] noted that higher flow rates cause formation of thick nanofiber and beads. The fiber diameter is increased because of reduction of charge density of fiber jet [266]. A bead is formed as the unstable jet is formed by the removal of the higher solution from the tip [267].

##### Ambient Environment

The effect of temperature and humidity on the electrospinning process cannot be ruled out. A lower temperature decreases the evaporation rate of the solvent, and eventually fiber diameter is decreased, as there is more time to be elongated before solidification. On the other hand, at a higher temperature, the diameter of the fiber increases, as the solution jet solidifies faster [257,268]. Moreover, the relative humidity can also have an impact on the fiber properties. Higher humidity can form a finer membrane. On the other hand, a lower humidity increases the fiber diameter [252,257,258,262].

Although extensive research has been done to understand the effect, there is significant space for additional research to reach a better understanding of the possible cause of bead formation and control of the fiber diameter. More comprehensive study is required to control the solution properties in order to understand the effect on electrospinning.

## 5. Future Directions

Research on the fabrication process of mixed-matrix membranes is ongoing, as they have been found very useful in different applications. Among all the mixed-matrix membranes, nanofibrous-type MMMs are now more popular due to their properties and efficiency. Among all the spinning processes, electrospinning has some great features like high speed, capability, and low cost, resulting in a highly porous patterned nanofibrous polymer membrane [269,270]. The electrospinning process can fabricate a membrane with a larger specific area with smaller pores and fibers within a diameter of 10 to 1000 nm [271]. These unique properties of electrospun nanofibrous membranes make them desirable for a wide range of applications [272], such as SiO_2_-incorporated electrospun SPEEK, which has been applied in a fuel cell [273]. Additionally, during the electrospinning process, it is easy to perform the ordering of the polymer, as well as the chain elongation. Considering all the mentioned characteristics of electrospinning, this process could be taken as the latest effective technology for the production of continuous, long-chain, mixed-matrix nanofibrous membranes using a combination of different polymers and nanomaterials for various applications on a large scale [246].

**Figure 17:**
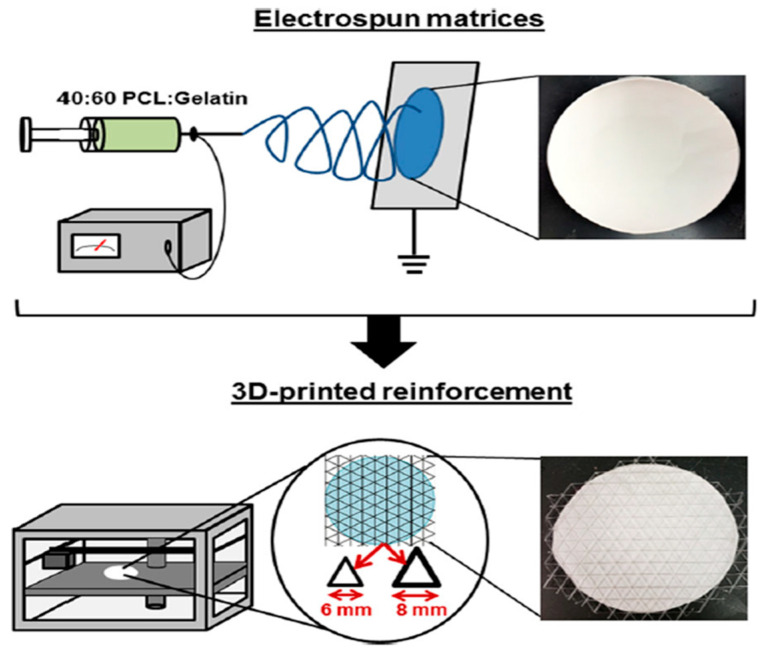
Fabrication of reinforced electrospun scaffolds. Electrospun scaffolds were produced from a 40:60 ratio of PCL:gelatin. The scaffolds were then placed in a 3D printer, and a PLA mesh was deposited onto one side of the scaffold. Two types of 3D-printed meshes were generated: one with a 6 mm distance between PLA struts, and the other with an 8 mm distance between struts (adapted with permission from [274]).

**Figure 18:**
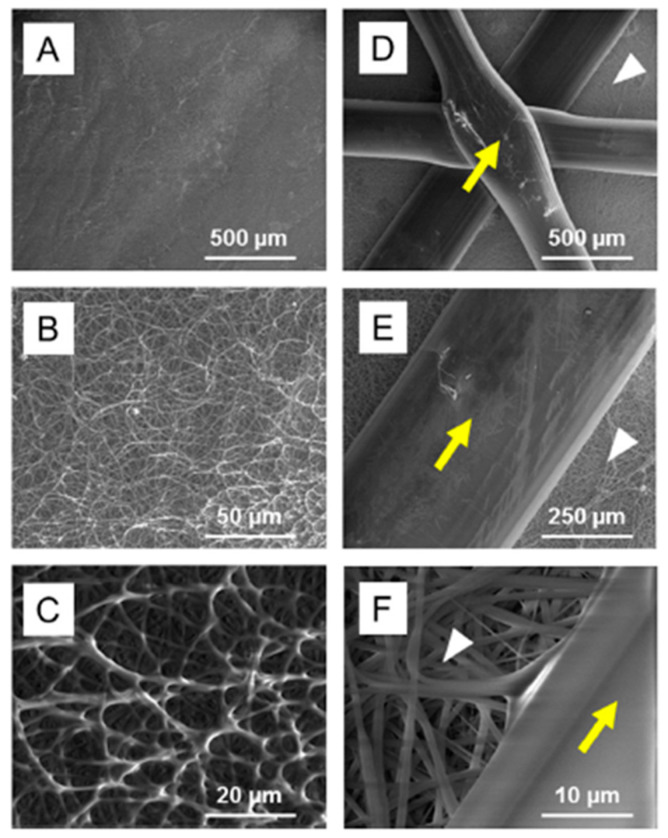
SEM images of reinforced electrospun scaffolds. (**A**–**C**) SEM images of the electrospun side of the reinforced scaffolds. The images show a uniform distribution of randomly oriented fibers. (**D**–**F**) SEM images of the 3D-printed side of the scaffolds. The high-magnification images (**F**) show that there is minimal damage to the electrospun fibers in the immediate vicinity of the 3D-printed PLA mesh. Yellow arrows depict the 3D-printed PLA. White arrowheads depict the PCL:gelatin scaffold (adapted with permission from [274]).

There is an opportunity for developing new technology combining 3D printing and electrospinning in the nanofiltration area, as has been done for biomedical applications (Figure 17). Recently, a 3D-printed mesh reinforcement on electrospun scaffolds was attempted, in which a poly (lactic acid) (PLA) mesh was 3D-printed into an electrospun poly(ε-caprolactone) (PCL) gelatin directly, resulting in better mechanical properties [274]. In Figure 18, the effect of 3D printing on the electrospun scaffold structure is clearly visible.

So, it can be concluded that this technique offers the same matrix-like structure with a higher mechanical strength of the electrospun membrane, and these modified and updated 3D-printed electrospun membranes could be used in a new range of membrane applications.

Nanocomposite materials; a combination of graphene, graphene oxides, or metal oxides such as ZnO, TiO_2,_ etc.; and magnetic nanoparticles, etc., could be better alternatives as filler materials in mixed-matrix membranes for various applications such as heavy metal removal, wastewater treatment, desalination, etc., as some previous research has shown that these types of nanocomposite particles excellently combine the properties that they exhibit individually [275,276]. The synthesis route of these nanocomposite particles is simple as well, using methods such as chemical mixing, chemical precipitation, sol–gel techniques, etc. [275,276,277].

Finally, the demand from the end user based on the applications is the main driving force for obtaining a good market value and establishing a better position in the total global membrane market, including pharmaceutical and biomedical, water filtration and wastewater treatment, textile and metalworking industries, chemicals and petrochemicals, food and beverages, etc. So, the demand for such fabrication technology is also at its peak, and a cost-effective and easier fabrication technology is desirable for bulk and industrial production.

## Figures and Tables

**Table 1:** Correlation between the surface roughness of commercial RO/NF membranes and their relative fluxes for the filtration of a 0.05 M NaCl solution containing 200 mg/L silica particles (0.10 µm); pH = 6.8. Flux-decline values determined for 10 L of permeate volume filtered *.

Membrane Type	Flux Decline, J/J_◦_, %	Average Roughness, nm	RMS Roughness, nm
Osmonics HL	13.9	10.1	12.8
Trisep X-20	38.3	33.4	41.6
Dow NF-70	46.9	43.3	56.5
HydranauticLFC-1	49.3	52.0	67.4

* Adapted with permission from [40].

**Table 2:** Basic properties of some membranes with their fabrication process.

Membrane-Fabrication Process	System	Driving Force	Membrane Properties	References
Phase inversion	PS/PVP/MXene nanosheets	Solvent and non-solvent interaction (NMP vs. water)	Porosity—79.4% Pore size—29 nm	[157]
Phase inversion	Polyimide-GO	Solvent and non-solvent interaction (NMP vs. water) and solvent exchange (2-propanol)	Porosity—65.3% Pore size—0.69 nm Surface Zeta Potential—37.6 MV	[13]
Electrospinning	PVDF	Voltage difference	Porosity—88% Electrolyte uptake—440% Conductivity—1.88 mS cm^−1^	[158]
Phase inversion	PVDF-PAN-SiO_2_	Solubility parameter difference, solvent and non-solvent miscibility	Conductivity—3.32 mS cm^−1^ Electrochemical stability—5 V Electrolyte uptake—246.8% Porosity—78.7%	[159]
Graft polymerization	PMMA–g-PE	Grafting PMMA, results in large uptake of electrolyte	Electrolyte uptake—350% Electrochemical stability—5 V Conductivity—1.3 mS cm^−1^	[160]
Electrospinning	Polyacrylonitrile/polyurethane	Voltage difference	Electrolyte uptake-776.1% Porosity—90.81% Conductivity—2.07 mS cm^−1^ Bulk resistance—1.2 Ω	[161]
Electrospinning	Poly(phthalazinone ether sulfone ketone)	Voltage difference	Electrolyte uptake—1210% Porosity—92% Conductivity—3.79 mS cm^−1^ Bulk resistance—1.2 Ω	[158]
Electrospinning	PS	Voltage difference	Fiber diameter—470 ± 150 nm Pore size—2.1 µm	[162]
Electrospinning and dip-coating	PEI/PVDF/x-PEGDA	Voltage difference for electrospun PEI/PVDF membrane and coating of x-PEGDA	Fracture Stress—12.1 MPa Pore size—2.56 μm Porosity—64.6% Electrolyte uptake—235.6% Conductivity—1.38 mS cm^−1^	[163]
Electrospinning	PEI/PVDF	Voltage difference	Fracture Stress—6.6 MPa Pore size—3.11 μm Porosity—83.5% Electrolyte uptake—492.8% Conductivity—1.03 mS cm^−1^	[163]
Electrospinning and coating	PE–PI–S	Voltage difference and coating	Porosity—60% Electrolyte uptake—400% Conductivity—1.34 mS cm^−1^	[164]
Electrospinning	PVDF-HFP	Voltage difference	Porosity—70% Electrolyte uptake—247% Conductivity—3.2 mS cm^−1^	[165]
Electrospinning	Trilayer (PVDF-HFP)/PVC/(PVDF-HFP)	Voltage difference	Porosity—62% Electrolyte uptake—230% Conductivity—1.58 mS cm^−1^	[165]
Electrospinning	PVDF/SiO_2_	Voltage difference	Porosity–85% Electrolyte uptake—646% Conductivity—7.47 mS cm^−1^	[166]
Electrospinning	Polyamic acid	Voltage difference	Pore size—800 nm Porosity—65.9% Electrolyte uptake—559%	[167]
Electrospinning	SiO_2_/nylon 6,6	Voltage difference	Porosity—77% Electrolyte uptake—360% Conductivity—3.8 mS cm^−1^	[168]
Electrospinning	PVDF-HFP/PEG/PEGDMA	Voltage difference	Electrolyte uptake—212% Porosity—71% Bulk resistance—0.94 Ω	[169]

**Table 3:** Merits and disadvantages of MMM fabrication processes.

MMM Fabrication Process	Merits	Disadvantages	References
Phase inversion	Simple process Economic	Difficult to produce a pinhole-free membrane	[170,171,172]
Interfacial polymerization	Produces a thin active layer with high flux of permeation and desired impurity rejection Defect-free Easy to scale up	Mainly depends on the properties of the monomer, so appropriate monomer selection is a crucial point	[173]
Electrospinning	Fabricates a membrane with high porosity, larger surface area, and outstanding pore interconnectivity. A mechanically stable membrane can be fabricated	Requires high voltage Uses a solvent Solubility issue for some polymers in a low-boiling-point solvent.	[174]
